# Involvement of the default mode network under varying levels of cognitive effort

**DOI:** 10.1038/s41598-022-10289-7

**Published:** 2022-04-15

**Authors:** Sarah Weber, André Aleman, Kenneth Hugdahl

**Affiliations:** 1 Department of Biological and Medical Psychology, University of Bergen, Bergen, Norway; 2 School of Health Sciences, Kristiania University College, Bergen, Norway; 3 Department of Biomedical Sciences of Cells and Systems, University of Groiningen, University Medical Center Groningen (UMCG), Groningen, The Netherlands; 4 Cognitive Neuroscience Center, University Medical Center Groningen (UMCG), Groningen, The Netherlands; 5 Division of Psychiatry, Haukeland University Hospital, Bergen, Norway; 6 Department of Radiology, Haukeland University Hospital, Bergen, Norway

**Keywords:** Cognitive neuroscience, Cognitive control

## Abstract

Everyday cognitive functioning is characterized by constant alternations between different modes of information processing, driven by constant fluctuations in environmental demands. At the neural level, this is realized through corresponding dynamic shifts in functional activation and network connectivity. A distinction is often made between resting and task processing and between task-negative and task-positive functional networks. The Default Mode Network (DMN) is classically considered as a resting state (i.e. task-negative) network, upregulated in the absence of cognitive demands. In contrast, task-positive networks have been labelled the Extrinsic Mode Network (EMN). We investigated changes in brain activation and functional network connectivity in an experimental situation of repeated alterations between levels of cognitive effort, following a block-design. Using fMRI and a classic Stroop paradigm, participants switched back and forth between periods of no effort (resting), low effort (word reading, i.e. automatic processing based on learned internal representations and rules) and high effort (color naming, i.e. cognitively controlled perceptual processing of specific features of external stimuli). Results showed an expected EMN-activation for task versus resting contrasts, and DMN-activation for rest versus task contrasts. The DMN was in addition more strongly activated during periods of low effort contrasted with high effort, suggesting a gradual up- and down-regulation of the DMN network, depending on the level of demand and the type of processing required. The often reported “anti-correlation” between DMN and EMN was strongest during periods of low effort, indicating intermittent contributions of both networks. Taken together, these results challenge the traditional view of the DMN as solely a task-negative network. Instead, both the EMN and DMN may contribute to low-effort cognitive processing. In contrast, periods of resting and high effort are dominated by the DMN and EMN, respectively.

## Introduction

The Default Mode Network (DMN) was discovered as a set of interconnected brain regions which are typically downregulated during the presence of external tasks or stimuli^1–3^. The DMN is therefore often labelled as a "task-negative" network, meaning that it is upregulated in the absence of demands for cognitive effort^4,5^. However, it is clear that sub-components, or nodes, of this network are present during cognitive processing, suggesting a more dynamic and complex role^6–8^. This opens up the question of how the DMN relates to what has been labelled "task-positive" networks^9,10^. Task-positive networks describe brain nodes and their functional connections that are upregulated in response to external stimulation and active task-processing. Apart from domain-specific networks, such as the visual or auditory networks, there are also non-specific task-positive networks which are activated across different cognitive domains^5,11–14^. Following the taxonomy introduced by Hugdahl et al.^13^, we will denote these task non-specific activations as an extrinsic mode network (EMN), in contrast to the DMN which is typically denoted as an intrinsic mode network^15,16^. The EMN has a morphological architecture with a fronto-temporo-parietal distribution, including the inferior and middle frontal gyri, inferior parietal lobule, supplementary motor area, and the inferior temporal gyrus^13^. It thus overlaps with what Fedorenko et al.^12^ called the cognitive flexibility network and Duncan^17^ called the multiple demand system. In addition, the EMN overlaps with domain-specific task-positive networks such as the salience, dorsal attention, and central executive networks^6,7,18^. Although EMN nodes correlate negatively with DMN nodes^5,14^, also called an anti-correlation^19,20^, there is evidence that the up- and down-regulation of task-negative and task-positive networks follows a gradual course when experimental demands abruptly alternate between rest and task-processing^5,14^. These findings suggest that the relationship between the DMN as a task-negative and the EMN as a task-positive network is more flexible than previously thought, with dynamic changes in the relative contributions of the two networks to brain functioning^21–23^. In everyday life, cognitive functioning is usually determined by frequent switching between different “tasks” with varying processing demands and with varying degrees of cognitive effort. Accordingly, brain functioning is likely to be characterized by a continuous shifting between varying contributions of different networks. For example, periods of highly effortful cognitive processing may engage the EMN more strongly than periods of lower cognitive effort^24^. While DMN activity has traditionally been viewed to interfere with performance on cognitive tasks^25^, we suggest that the DMN may also contribute positively to performance under certain circumstances. On the one hand, DMN involvement has been established for self-referential processing, such as autobiographical memory and imagining one’s future, and for theory of mind reasoning^26^. On the other hand, a more recent body of evidence has established a role for the DMN in certain aspects of perceptual tasks (see^27^ for a review). For example, it has been argued that the DMN is more strongly activated by tasks that require low-effort, automatic processing than by high-effort tasks. Provost et al.^28^ (see also^20^) used the Wisconsin Card Sorting task, with a repetitive, low-effort condition where subjects had to sort the cards according to the same rule over a longer period of time, and a high-effort condition where they had to switch between sorting rules. The results showed increased DMN activation in the former compared to the latter condition, suggesting a contribution of DMN to low-, but not high-effort cognitive processing. Similarly, Vatansever et al.^29^ divided performance on the Wisconsin Card Sorting task into a high-effort learning phase where participants had to work out the correct sorting rule, and a low-effort phase where they only had to apply this rule over several trials. DMN activity was increased in the first phase compared to the second phase of the task. The authors concluded that the DMN might be crucial for rapid automatic information processing in situations of predictable cognitive demands.

Other researchers have shown that the DMN is consistently involved in tasks that require memory guided decision making, i.e. using information from previous trials to make judgements about visual stimuli. When performing a 1-back task with pictures of either shapes or objects, participants showed activity in typical DMN regions, such as the posterior cingulate cortex (PCC), angular gyri and lateral temporal cortex^30–32^. Interestingly, these DMN regions were more active during the 1-back condition than during a 0-back control condition in which participants had to make same/different-judgement on the stimuli presented. Behavioral data showed that performance was better in the 0-back task than in the 1-back task, indicating lower cognitive effort in the former than in the latter task. These findings speak against previously discussed interpretations of level of effort being the driving force for DMN involvement in task processing. Instead, the research on memory guided decision making might suggest that it is the use of internal stimulus representations that activates the DMN. This view is further supported by studies that show a relationship between DMN activity and the level of detail in internal ongoing thought while performing a cognitive task^33,34^. In the same way, the previously discussed DMN activity during the WCST^28,29^ might be driven by the use of internal task-related representations in the form of learned sorting rules from previous trials, instead of driven by low cognitive effort. In conclusion, there is now overwhelming evidence that the DMN is not simply a task-negative network that is activated during periods of rest, but instead actively contributes to the performance of externally guided task under some circumstances. Although it is still difficult to link the DMN to a specific cognitive process, it seems like it might be related to internal mental representations that are important for task performance and are activated by external demands, for example in the form of self-referential mentalizing or memory-guided decision making. One explanation for this role of the DMN might be its location, since it consists of regions that relatively far removed from sensory and motor regions of the cortex, which might enable it to act more independently of the direct environment than other regions^27^.

We aimed to follow-up earlier studies by investigating different modes of cognitive processing, with a focus on interactions between the DMN and EMN networks by combining analyses of BOLD activation and functional connectivity. Cognitive processing was manipulated in an ON–OFF block-design based on the Stroop task^35^ with three experimental conditions. The three conditions differed in the amount of cognitive effort and in the type of processing that was required to perform them. In a *high-effort* condition, participants named the ink color of the color words presented, irrespective of the words’ meaning. This condition is cognitively demanding since it requires active inhibition of the interfering meaning of the words, in addition to redirection of attention. In addition, the condition has a strong external focus because it relies heavily on the perceptual processing of the color of the stimulus. In a *low-effort* condition, stimulus presentation was identical to the high-effort condition, but participants were instructed to read the color words irrespective of the color they were presented in. Since reading is an overlearned response, this condition is characterized by automatic, low-effort cognitive processing. The condition also requires combined externally and internally focused processing. On the one hand, visual features of the stimulus have to be processed for letter recognition. On the other hand, these visual features have to be combined with learned internal representations of familiar letters and words. These learned representations might be used in the same way as internal stimulus representations from previous trials in memory guided decision making^30–32^. In a third *no-effort* condition, participants passively viewed a fixation cross, with no words presented. This condition was free of external demands and allowed for task-independent cognition. We predicted that the EMN would be more activated during the high-effort ink-naming condition that combined perceptual processes with cognitive control, compared to the low-effort word reading condition, and during low-effort word reading compared to no-effort resting. Similarly, we predicted that the DMN would be more activated during resting, characterized by lack of effort and internally guided processing, compared to word reading, characterized by medium effort and a combination of external stimulus processing and use of internal representations. Furthermore, DMN activity should be higher during the low-effort word reading compared to the high-effort ink naming which relies most heavily on controlled perceptual processing. With regard to functional connectivity, most previous research in this context has focused on comparisons between task and rest (e.g.^21,22^). We expected differential DMN connectivity when comparing high- and low effort conditions, with increased negative connectivity between then DMN and EMN under low-effort processing compared to high-effort processing (cf.^29^).

## Methods

### Participants

44 healthy adults were recruited through flyers in the university and university hospital environment. The mean age was 26.18 years (SD = 4.37, range 21–38 years). 25 participants were female. All participants were right-handed native Norwegian speakers and reported normal color vision and no history of dyslexia or psychiatric or neurological conditions. Participants gave informed consent prior to participation. The study was conducted according to the Declaration of Helsinki and had received approval from the Norwegian ethics authorities (Regional Committees for Medical and Health Research Ethics (REK), project reference 2017/2452).

### Materials and procedure

#### Stroop task

Participants performed a version of the Stroop paradigm^28^ that included three different levels of cognitive effort. In the high-effort condition, participants were asked to name the print color of the words that were shown, ignoring the meaning of the word. In the low-effort condition, participants were asked to read the color words out loud. An additional no-effort condition consisted of passive viewing of a fixation cross. Participants were instructed of the procedure for the three conditions and given examples before going into the MR scanner.

The three conditions were presented in alternating 30-s blocks (ABCACB…), each starting with a 3-s prompt screen that informed about which condition was to follow. There were eight blocks per condition. Each of the two task-blocks consisted of 18 stimuli presented in pseudorandom order with no more than three consecutive repetitions of identical stimuli, same color word or same printing color. The stimuli were four Norwegian color-words "blå" (blue), "grønn" (green), "gul" (yellow), and "rød" (red) written in blue, green, yellow or red ink in the center of the LCD screen, on black background. Presentation time was 250 ms with a 1500 ms gap between words in which participants gave their response verbally. Stimuli were presented with the E-prime software (version 2.0; https://pstnet.com/products/e-prime/) and viewed through MR-compatible LCD goggles which were adjusted to individual eyesight. Visibility was verified by each participant before the experiment started. Participants’ responses were recorded through an MR-compatible microphone and recording device which was attached to the head coil.

#### MRI scanning parameters

Data were acquired at the Haukeland University Hospital in Bergen, Norway, on a Siemens Prisma 3 T MR scanner. A structural T1-weighted scan was acquired with a seven-minute sequence, using the following parameters: repetition time (TR) = 1800 ms, echo time (TE) = 2.28 ms, flip angle = 8°, voxel size = 1 × 1 × 1 cm, field of view (FOV) = 256 × 256. During the Stroop task, a functional EPI-scan was acquired with the following parameters: TR = 2000 ms, TE = 30 ms, flip angle = 90°, voxel size = 3.6 × 3.6 × 3.6 cm, 34 slices (10% gap between slices), FOV = 1380 × 1380. The scan lasted approximately 15 min. In total, participants spent approximately 40 min in the scanner, including resting-state and MR spectroscopy sequences which is not reported in the current paper.

#### fMRI preprocessing and analysis

EPI-data were preprocessed with the SPM-software (SPM12, https://www.fil.ion.ucl.ac.uk/spm/software/spm12/), using a standard preprocessing pipeline with realignment of functional images, coregistration with the T1 image, normalization into Montreal Neurological Institute (MNI) standard space, and smoothing with a Gaussian kernel of FWHM 6 mm. Parameters from the SPM motion correction procedure were analyzed in order to check that there was no excessive head motion. None of the participants had movements of greater than one voxel along any of the three axes.

Brain activity analyses were conducted in SPM with a classic block-design model containing rest-blocks as an implicit baseline, using block onsets convolved with a double-gamma hemodynamic response function to define the different conditions. Contrasts between the three conditions were calculated to reflect differences in cognitive effort: low effort (word reading)—no effort (rest condition); high effort (ink color naming)—no effort (rest condition), high effort (ink color naming)—low effort (word reading), plus the reverse of these contrasts. All results were FEW-corrected at p = 0.008 (Bonferroni-corrected for the six contrasts).

Functional connectivity analyses were conducted in the Conn-toolbox (version v.17.f http://www.nitrc.org/projects/conn). Preprocessed data first went through the Conn-toolbox default denoising pipeline, where motion parameters and their first derivatives as well as the BOLD-signal from white matter and cerebrospinal fluid masks (first five principle components for each) were regressed out. Linear detrending and a band-pass filter of 0.008–0.09 Hz was applied. Subsequently, seed-based functional connectivity analyses were conducted, and high-effort blocks were contrasted with low-effort blocks. Seed regions were derived from activation clusters from the block-activation analyses, resulting in a DMN seed and an EMN seed (see results section "Functional connectivity for different levels of cognitive processing" below for further details). All results were corrected for multiple comparisons using Conn’s default FDR-cluster correction with a voxel-level p < 0.001 and cluster-level p = 0.025 (Bonferroni-corrected for the two seeds).

## Results

### Behavioral results: stroop performance

A paired-sample t-test was performed to compare performance between the low- and high-effort conditions. Accuracy of responses was significantly lower in the high-effort condition compared to the low-effort condition (*M* = 96.42%, *SD* = 2.81, and *M* = 99.11, *SD* = 1.09, respectively), *t*(43) = 7.13, *p* < 0.001.

### fMRI results

#### fMRI activation for different levels of cognitive effort

**Figure 1:**
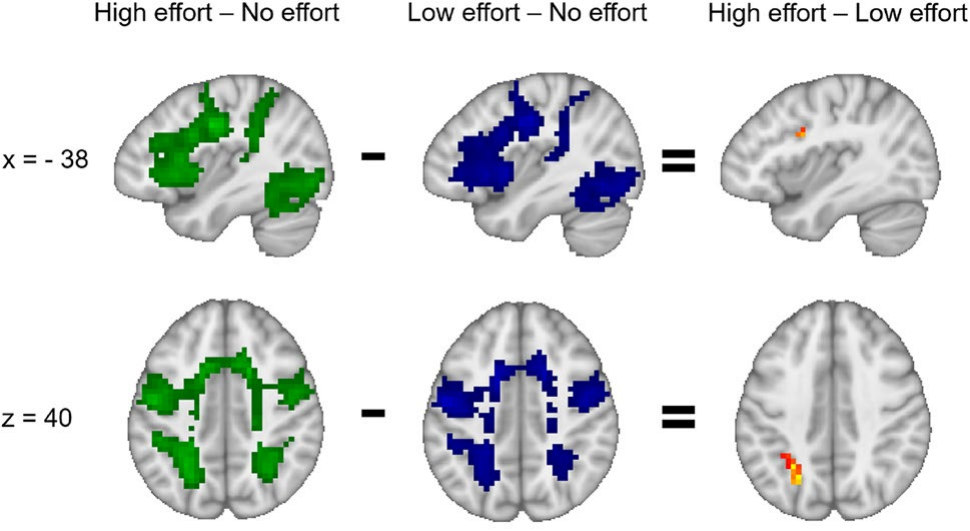
More intense activation in typical EMN areas for the comparisons of high effort versus rest (green), low effort versus rest (blue) and high effort versus low effort (yellow/red). The left side of the axial images corresponds to the left hemisphere.

Comparing the high- and low-effort tasks to the resting condition resulted in bilateral activation of frontal/precentral, parietal, occipital and superior temporal areas (Fig. 1). Activations for these two contrasts showed however extensive overlap. Comparing high-effort to low-effort directly only resulted in small activation clusters, with stronger activity for the high-effort condition in the left superior parietal gyrus, left precentral/middle frontal gyrus and supplementary motor area (Fig. 1). Details on cluster statistics for the three contrasts can be found in Supplementary Tables S1–S3.

**Figure 2:**
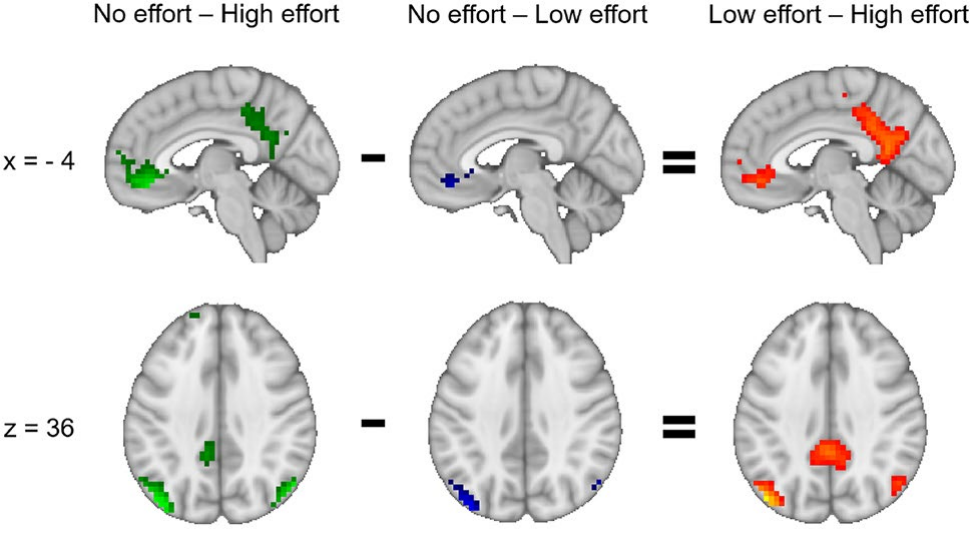
More intense activation in typical DMN areas for the comparisons of rest versus high effort (green), rest versus low effort (blue) and low effort versus high effort (yellow/red). The left side of the axial images corresponds to the left hemisphere.

Comparing the resting condition to high- and low-effort, respectively, resulted in significant activation in hub-areas of the DMN, in the inferior parietal cortices and in the anterior cingulate cortex (ACC) for both contrasts, and additionally in the posterior cingulate cortex (PCC) for the resting versus high-effort condition only (Fig. 2). Comparing the two task-conditions with each other showed significantly higher activation in the parietal cortex, ACC and PCC for the low- versus high-effort contrast (Fig. 2). Details on cluster statistics for the three contrasts can be found in Supplementary Tables S4-S6.

#### Functional connectivity for different levels of cognitive effort

The aim was to investigate functional connectivity between EMN and DMN areas that were susceptible to manipulations of cognitive effort. Therefore, activation clusters from the contrasts (high effort–low effort) as well as (low effort–high effort) were used as seeds for the whole-brain connectivity analyses. This resulted in one seed comprising EMN areas (Fig. 1, upper and lower right panel) and one seed comprising DMN areas (Fig. 2, upper and lower right panel). For each of the two seeds, connectivity during low-effort blocks was compared to connectivity during high-effort blocks.

##### EMN-seeded functional connectivity

**Figure 3:**
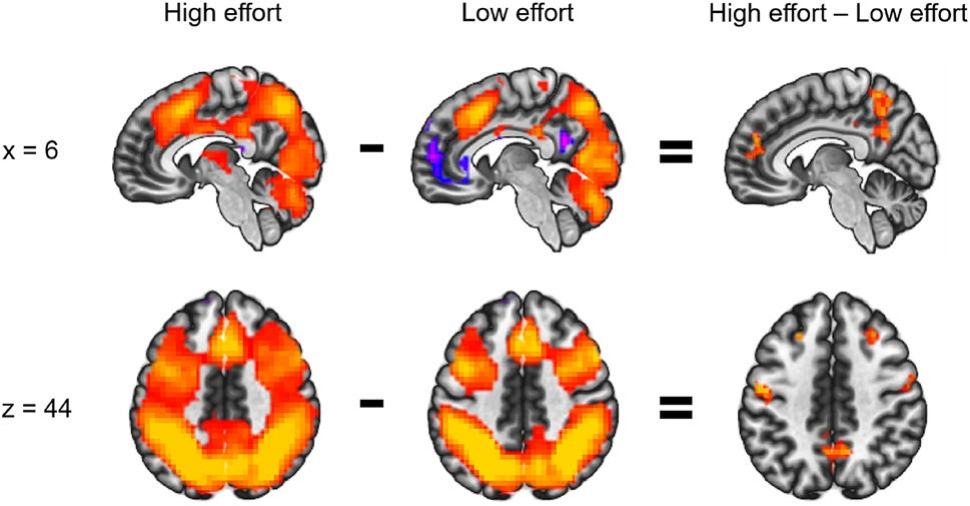
Sagittal and axial view of clusters that showed significant functional connectivity with the EMN seed (shown in Fig. 1, right panel) during high effort (ink condition), during low effort (word condition), and differences when comparing the two conditions. Positive connectivity values are shown in red/orange and negative connectivity values in blue/purple. In the direct comparison, red indicates more positive connectivity values for high than low effort. The left side of the axial images corresponds to the left hemisphere.

During high effort compared to low effort, the EMN seed showed significantly stronger connectivity with other areas of the EMN, namely superior and middle frontal gyrus, pre-/postcentral gyrus, SMA and superior parietal cortex. Post-hoc analyses for low-effort and high-effort blocks separately showed positive connectivity during both conditions, but this positive connectivity was stronger during high-effort blocks. Furthermore, EMN-connectivity with the ACC and the PCC was significantly different during high effort compared to low effort, there was a significant negative connectivity between these regions during low-effort blocks which was not present during high-effort blocks (Fig. 3). Statistics details for the EMN-seeded connectivity results can be found in Supplementary Table S7.

##### DMN-seeded functional connectivity

**Figure 4:**
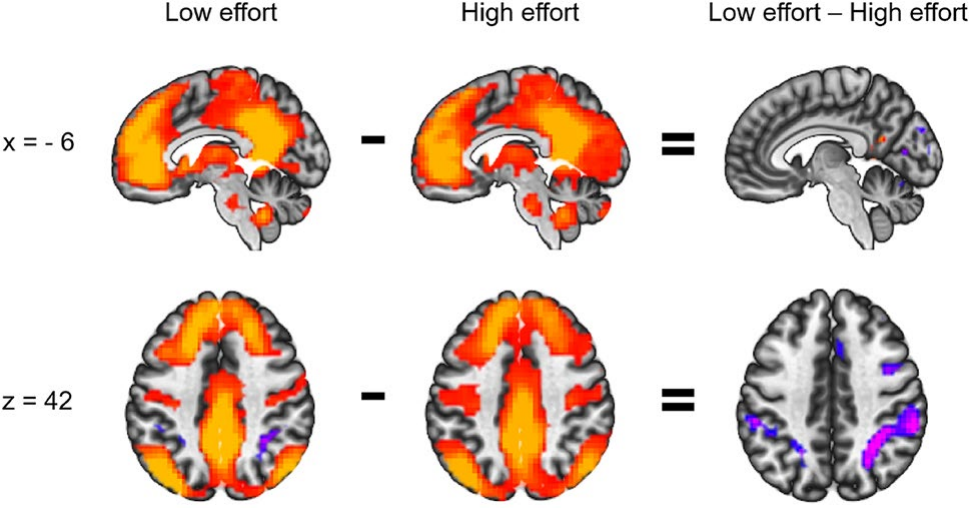
Sagittal and axial view of clusters that showed significant functional connectivity with the DMN seed (shown in Fig. 2, right panel) during low effort (word condition), during high effort (ink condition), and differences when comparing the two conditions. Positive connectivity values are shown in red/orange and negative connectivity values in blue/purple. In the direct comparison, red indicates more positive connectivity values and blue more negative values for low effort than high effort. The left side of the axial images corresponds to the left hemisphere.

The DMN-seed showed significantly stronger connectivity with a small cluster in the PCC during low-effort compared to high-effort blocks. Connectivity was positive for both conditions separately but stronger for low-effort blocks. Furthermore, the DMN-seed showed significantly stronger negative connectivity with the SMA, superior and middle frontal gyrus, precentral gyrus, IFG and bilateral superior parietal cortex during low-effort compared to high-effort blocks. Post-hoc analyses showed that the difference in connectivity stemmed from a negative connectivity during low-effort blocks, not present during high-effort blocks (Fig. 4). In order to test whether the low-effort condition was the only condition with an anti-correlation between the networks, we additionally calculated connectivity values for the rest condition, even though this was not part of the main focus of this study. The results showed some anti-correlations between the DMN-seed and parietal EMN areas, but connectivity was less extensive and less strong than for the low-effort condition (see Supplementary Fig. 1). Statistics details on the DMN-seeded connectivity results can be found in Supplementary Table S8.

## Discussion

The current study aimed to investigate two cortical networks, DMN and EMN, under conditions of varying cognitive processing. Functional activation as well as connectivity was examined while participants switched between processing task blocks with a high-effort condition that required cognitively controlled perceptual processing, a low-effort condition that required automatic processing based on learned internal representations and rules, and a no-effort condition with passive resting. The type of cognitive processing modulated activation in both networks, as well as connectivity between the networks. As expected, conditions of cognitive effort compared to rest activated fronto-parietal areas that have previously been found to be activated across a variety of cognitive tasks and together constitute the EMN^12–14,17^. In line with previous research^21,29^ frontal and parietal parts of the EMN were also significantly more activated during high-effort perceptual processing compared to low-effort rule-based processing, although the extent of these activation differences was relatively small. This finding suggests that the EMN is activated as soon as external stimulation or task demands are present, relatively independent of the level of effort involved, following a threshold mechanism of activation.

The areas that showed significantly more activation during resting compared with both task conditions have previously been described to constitute the “core DMN”^1,36^. In line with recent findings^29^, DMN areas in the present study were also more active during the low-effort rule-based processing compared to the high-effort perceptual condition, and this difference was even more pronounced than the difference between low-effort and resting. Thus, contrary to the traditional view of the DMN as a unique task-negative network which is down-regulated in the face of cognitive demands, or may even interfere with cognitive performance, these results indicate a more nuanced up- and downregulation depending on the amount of cognitive effort and the type of processing required. The largest difference between low and high effort was found in the PCC, which was downregulated only during high effort compared to resting, but not during low effort compared to resting. The PCC has often been described as a central hub for the DMN^1,36,37^. It may therefore be the “last man standing” when other nodes of the DMN are downregulated. Gradual downregulation of PCC activity with increasing task difficulty has previously been shown during semantic processing^38^. Interestingly, these decreases in PCC activity were accompanied by increases in its functional connectivity with task-positive frontal regions, speaking to its central role in cognition. Taken together, these results suggest that up- and downregulation of the EMN and DMN is tuned in a way that the EMN is activated in a threshold-manner whenever there are requirements for controlled processing, even if the load is minimal, and additional activation or deactivation of the DMN is a regulatory parameter which is dependent on the degree of effort and the type of processing required.

The areas of the DMN and the EMN that showed significant differential activation for low and high effort in the block-analysis, also showed differential effort-dependent connectivity patterns, when these areas were used as seed regions. The DMN showed stronger positive connectivity with its own core region, the PCC, during low effort compared to high effort. Increased connectivity between different DMN nodes has previously been found when comparing resting to cognitive processing^21,22^, and when comparing low-effort to high-effort processing^27^. Strong connectivity between DMN nodes has been interpreted as network stability and is positively correlated with cognitive performance^39^ and negatively correlated with for example age^40^. EMN-seeded connectivity was also modulated by cognitive effort. Comparing high- to low-effort processing, the EMN showed stronger positive connectivity with frontal and parietal areas which are typically associated with attention and cognitive control^13,41^, thus showing increased within-connectivity between EMN nodes when task-demands and effort is high. Such increases could guide effortful cognitive processing, as suggested by previous findings of positive correlations between strong within-EMN connectivity and working memory load, as well as individual performance under conditions of high load^42^.

Cognitive effort did not only modulate connectivity within networks but also connectivity between the two networks. The anti-correlation that has often been reported for task-negative and task-positive networks^19,20^, was significantly stronger during low-effort processing than during high-effort processing. In fact, post-hoc analyses for the two levels of cognitive effort separately showed that there was a significant anti-correlation between the DMN and EMN only for the low-effort condition. This was true when using both the DMN- and the EMN-seed, which supports the robustness of the finding. In the rest condition, anti-correlation was marginally present but less pronounced in extent and strength than during low effort. These results are in line with previously reported patterns of DMN connectivity^29^, although these authors failed to find equivalent results when using a seed located in the task-positive network. A possible explanation for this discrepancy could be the exact location of the task-positive seed in the two studies. While Vatansever et al.^29^ used a frontal eye field ROI as a seed, a network-seed involving all areas with significantly greater activation for high compared to low effort was used in the present study. It seems that the whole network seed, rather than a single ROI seed, collectively contributes to differences in functional connectivity when levels of cognitive effort vary.

Overall, the present results indicate that both the EMN and DMN contribute to low-effort cognitive processing, in the sense that the EMN is upregulated during such periods, but the DMN is not fully downregulated. The anti-correlation between the two networks during these periods could suggest that their contributions happen intermittently. That is, periods of EMN dominance and DMN downregulation are alternated with states of DMN dominance and EMN downregulation, rather than a continuous parallel upregulation of both networks. In contrasts, periods of resting and periods of high effort are uniquely dominated by activation of the DMN and EMN, respectively. An interpretation of this is that under conditions of high cognitive effort, activation in the two networks is independent of each other, which in turn could explain why there is weaker anti-correlation between the networks during such periods. We interpret this that under conditions of high to extreme cognitive effort, the task-positive EMN is so strongly up-regulated that it completely dominates and supersedes any dynamics related to the DMN. This is not the case during low-effort conditions, where both networks contribute, which in turn contributes to the anti-correlation between them.

Because our low-effort task concerned an automatized process (reading), performance was very high, precluding calculation of correlations with DMN involvement due to a ceiling effect. Such a correlation would lend direct support for positive contributions of the DMN to cognitive processing in this condition. An alternative explanation would be that part of the DMN activation during low-effort processing is related to task-independent thought, which is more prominent when task demands are lower^43^. A study investigating different levels of working memory performance reported decreased engagement and responsiveness of the DMN in pain patients but performance was intact (i.e. not different from healthy subjects), without demonstrable compensatory neural recruitment^44^. The authors concluded that a responsive DMN might not be needed for successful cognitive performance. On the other hand, other studies have shown positive relationships between DMN activity and the degree of detail in representations of task-relevant information during active task states^34^. In addition, positive correlations between task performance and DMN functional connectivity with task-positive regions^29^ suggest a beneficial role of DMN involvement for cognitive performance.

The current results add to a growing body of evidence for an active role of the DMN in cognitive processing and a dynamic relationship with task-positive regions. However, as the present study has shown, the degree of such involvement is dependent on the specific cognitive requirements at any moment in time. In addition, the exact nature of the DMN’s contribution to cognition is still unclear. Sormaz et al.^34^ reported a contribution of the DMN to ongoing cognition, extending beyond task-unrelated processing, which is at odds with the task-negative view of the DMN. Using self-report data describing levels of detail of experience, relationship to a task, and emotional qualities they found that during periods of active working-memory maintenance, activity within the DMN was associated with the level of detail in ongoing thought. Similarly, Murphy et al.^33^ found that participants’ tendency for detail in ongoing thought was associated specifically with PCC activity during a self-referential task. Research on memory guided decision making also suggest that DMN activity is relevant for task-related information processing, here in the form of internal stimulus representations from previous trials^30–32^. Similarly, DMN activity during the WCST might be driven by internal representations of sorting rules developed in previous trials^28,29^. All of these tasks require a combination of external stimuli as well as internal representations, which could explain the activation of EMN as well as DMN regions. That is, external task demands activate the EMN, whereas the internal representations that are needed for these types of tasks activate the DMN. In the current study, the reading condition required the combination of externally presented words and internal overlearned representations of these words. The current results might therefore extend the existing literature by showing that the DMN’s role in processing internal task-relevant information does not only apply to recently (i.e. in the previous trial) acquired information, but also more generally to representations that are stored in long-term memory. In contrast, other researchers have suggested that in particular the PCC as a highly connected core hub is involved in controlling cognition by monitoring changes in cognitive demands and integrating information from different networks in order to adjust behavior accordingly^45,46^. More recently, others have suggested that the PCC’s role is not only related to monitoring but also predicting cognitive demands in the environment^45,47^. Such predictions would be easier during periods of highly rule-based automatic cognitive processing, which would explain greater engagement of the DMN during these periods.

In summary, we found that large-scale network dynamics during task-processing is dependent on the load of cognitive effort and the type of cognitive processing. During low-effort conditions that require the use of internal mental representations, the DMN and EMN both contribute to the observed activation patterns, while activations in situations characterized by high effort and resting are separately driven by the EMN and DMN, respectively.

## Supplementary Information

Supplementary Information 1.

Supplementary Information 2.

## References

[1] Buckner RL, Andrews-Hanna JR, Schacter DL (2008). The brain’s default network anatomy, function, and relevance to disease. Ann. N. Y. Acad. Sci..

[2] Raichle ME, MacLeod AM, Snyder AZ, Powers WJ, Gusnard DA, Shulman GL (2001). A default mode of brain function. Proc. Natl. Acad. Sci..

[3] Shulman GL, Corbetta M, Buckner RL, Fiez JA, Miezin FM, Raichle ME, Petersen SE (1997). Common blood flow changes across visual tasks: I. Increases in subcortical structures and cerebellum but not in nonvisual cortex. J. Cogn. Neurosci..

[4] Anticevic A, Cole MW, Murray JD, Corlett PR, Wang XJ, Krystal JH (2012). The role of default network deactivation in cognition and disease. Trends Cogn. Sci..

[5] Hugdahl K, Kazimierczak K, Beresniewicz J, Kompus K, Westerhausen R, Ersland L, Specht K (2019). Dynamic up- and down-regulation of the default (DMN) and extrinsic (EMN) mode networks during alternating task-on and task-off periods. PLoS ONE.

[6] Bressler SL, Menon V (2010). Large-scale brain networks in cognition: emerging methods and principles. Trends Cogn. Sci..

[7] Greicius MD, Krasnow B, Reiss AL, Menon V (2003). Functional connectivity in the resting brain: A network analysis of the default mode hypothesis. Proc. Natl. Acad. Sci..

[8] Ossandón T, Jerbi K, Vidal JR, Bayle DJ, Henaff M-A, Jung J, Minotti L, Bertrand O, Kahane P, Lachaux J-P (2011). Transient suppression of broadband gamma power in the default-mode network is correlated with task complexity and subject performance. J. Neurosci..

[9] Uddin LQ, Kelly AMC, Biswal BB, Castellanos FX, Milham MP (2009). Functional connectivity of default mode network components: correlation, anticorrelation, and causality. Hum. Brain Mapp..

[10] Yamashita M, Shimokawa T, Takahashi S, Yamada S, Terada M, Ukai S, Tanemura R (2020). Cognitive functions relating to aberrant interactions between task-positive and task-negative networks: Resting fMRI study of patients with schizophrenia. Appl. Neuropsychol. Adult.

[11] Duncan J, Owen AM (2000). Common regions of the human frontal lobe recruited by diverse cognitive demands. Trends Neurosci..

[12] Fedorenko E, Duncan J, Kanwisher N (2013). Broad domain generality in focal regions of frontal and parietal cortex. Proc. Natl. Acad. Sci..

[13] Hugdahl K, Raichle ME, Mitra A, Specht K (2015). On the existence of a generalized non-specific task-dependent network. Front. Hum. Neurosci..

[14] Riemer F, Grüner R, Beresniewicz J, Kazimierczak K, Ersland L, Hugdahl K (2020). Dynamic switching between intrinsic and extrinsic mode networks as demands change from passive to active processing. Sci. Rep..

[15] Fox MD, Zhang D, Snyder AZ, Raichle ME (2009). The global signal and observed anticorrelated resting state brain networks. J. Neurophysiol..

[16] Raichle ME (2010). Two views of brain function. Trends Cogn. Sci..

[17] Duncan J (2013). The structure of cognition: Attentional episodes in mind and brain. Neuron.

[18] Corbetta M, Patel G, Shulman GL (2008). The reorienting system of the human brain: From environment to theory of mind. Neuron.

[19] Fox MD, Snyder AZ, Vincent JL, Corbetta M, van Essen DC, Raichle ME (2005). The human brain is intrinsically organized into dynamic, anticorrelated functional networks. Proc. Natl. Acad. Sci..

[20] Provost JS, Monchi O (2015). Exploration of the dynamics between brain regions associated with the default-mode network and frontostriatal pathway with regards to task familiarity. Eur. J. Neurosci..

[21] Elton A, Gao W (2015). Task-positive functional connectivity of the default mode network transcends task domain. J. Cogn. Neurosci..

[22] Gao W, Gilmore JH, Alcauter S, Lin W (2013). The dynamic reorganization of the default-mode network during a visual classification task. Front. Syst. Neurosci..

[23] Piccoli T, Valente G, Linden DE, Re M, Esposito F, Sack AT, Salle FD (2015). The default mode network and the working memory network are not anti-correlated during all phases of a working memory task. PLoS ONE.

[24] McKiernan KA, Kaufman JN, Kucera-Thompson J, Binder JR (2003). A parametric manipulation of factors affecting task-induced deactivation in functional neuroimaging. J. Cogn. Neurosci..

[25] Nathan Spreng R (2012). The fallacy of a “task-negative” network. Front. Psychol..

[26] Spreng RN, Grady CL (2010). Patterns of brain activity supporting autobiographical memory, prospection, and theory of mind, and their relationship to the default mode network. J. Cogn. Neurosci..

[27] Smallwood J, Bernhardt BC, Leech R, Bzdok D, Jefferies E, Margulies DS (2021). The default mode network in cognition: A topographical perspective. Nat. Rev. Neurosci..

[28] Provost J-S, Petrides M, Simard F, Monchi O (2012). Investigating the long-lasting residual effect of a set shift on frontostriatal activity. Cereb. Cortex.

[29] Vatansever D, Menon DK, Stamatakis EA (2017). Default mode contributions to automated information processing. Proc. Natl. Acad. Sci..

[30] Konishi M, McLaren DG, Engen H, Smallwood J (2015). Shaped by the past: the default mode network supports cognition that is independent of immediate perceptual input. PLoS ONE.

[31] Murphy C, Jefferies E, Rueschemeyer SA, Sormaz M, Wang HT, Margulies DS, Smallwood J (2018). Distant from input: Evidence of regions within the default mode network supporting perceptually-decoupled and conceptually-guided cognition. Neuroimage.

[32] Murphy C, Wang HT, Konu D, Lowndes R, Margulies DS, Jefferies E, Smallwood J (2019). Modes of operation: A topographic neural gradient supporting stimulus dependent and independent cognition. Neuroimage.

[33] Murphy C, Poerio G, Sormaz M, Wang HT, Vatansever D, Allen M, Smallwood J (2019). Hello, is that me you are looking for? A re-examination of the role of the DMN in social and self relevant aspects of off-task thought. PLoS ONE.

[34] Sormaz M, Murphy C, Wang HT, Hymers M, Karapanagiotidis T, Poerio G, Smallwood J (2018). Default mode network can support the level of detail in experience during active task states. Proc. Natl. Acad. Sci..

[35] Stroop JR (1935). Studies of interference in serial verbal reactions. J. Exp. Psychol..

[36] Christoff K, Fox K, Spreng RN, Andrews-Hanna JR (2016). Mind-wandering as spontaneous thought: A dynamic framework. Nat. Rev. Neurosci..

[37] Raichle ME (2015). The Brain’s default mode network. Annu. Rev. Neurosci..

[38] Krieger-Redwood K, Jefferies E, Karapanagiotidis T, Seymour R, Nunes A, Ang JWA, Smallwood J (2016). Down but not out in posterior cingulate cortex: Deactivation yet functional coupling with prefrontal cortex during demanding semantic cognition. Neuroimage.

[39] Mak LE, Minuzzi L, MacQueen G, Hall G, Kennedy SH, Milev R (2017). The default mode network in healthy individuals: A systematic review and meta-analysis. Brain Connect..

[40] Grady C, Sarraf S, Saverino C, Campbell K (2016). Age differences in the functional interactions among the default, frontoparietal control, and dorsal attention networks. Neurobiol. Aging.

[41] Passingham R (2021). Understanding the Prefrontal Cortex: Selective Advantage, Connectivity, and Neural Operations.

[42] Liang X, Zou Q, He Y, Yang Y (2016). Topologically reorganized connectivity architecture of default-mode, executive-control, and salience networks across working memory task loads. Cereb. Cortex.

[43] Smallwood J, Nind L, O’Connor RC (2009). When is your head at? An exploration of the factors associated with the temporal focus of the wandering mind. Conscious. Cogn..

[44] Čeko M, Gracely JL, Fitzcharles MA, Seminowicz DA, Schweinhardt P, Bushnell MC (2015). Is a responsive default mode network required for successful working memory task performance?. J. Neurosci..

[45] Dohmatob E, Dumas G, Bzdok D (2020). Dark control: The default mode network as a reinforcement learning agent. Hum. Brain Mapp..

[46] Leech R, Braga R, Sharp DJ (2012). Echoes of the brain within the posterior cingulate cortex. J. Neurosci..

[47] Araña-Oiarbide, G., Daws, R. E., Lorenz, R., Violante, I. R. & Hampshire, A. Preferential activation of the posterior default-mode network with sequentially predictable task switches. Preprint at https://www.biorxiv.org/content/10.1101/2020.07.29.223180v1?rss=1 (2020). doi:10.1101/2020.07.29.223180

