# Supplementary figures and images for: Involvement of the default mode network under varying levels of cognitive effort
Source: Sci Rep. 2022 Apr 15;12:6303. doi: 10.1038/s41598-022-10289-7 (PMC9012747; doi:10.1038/s41598-022-10289-7)

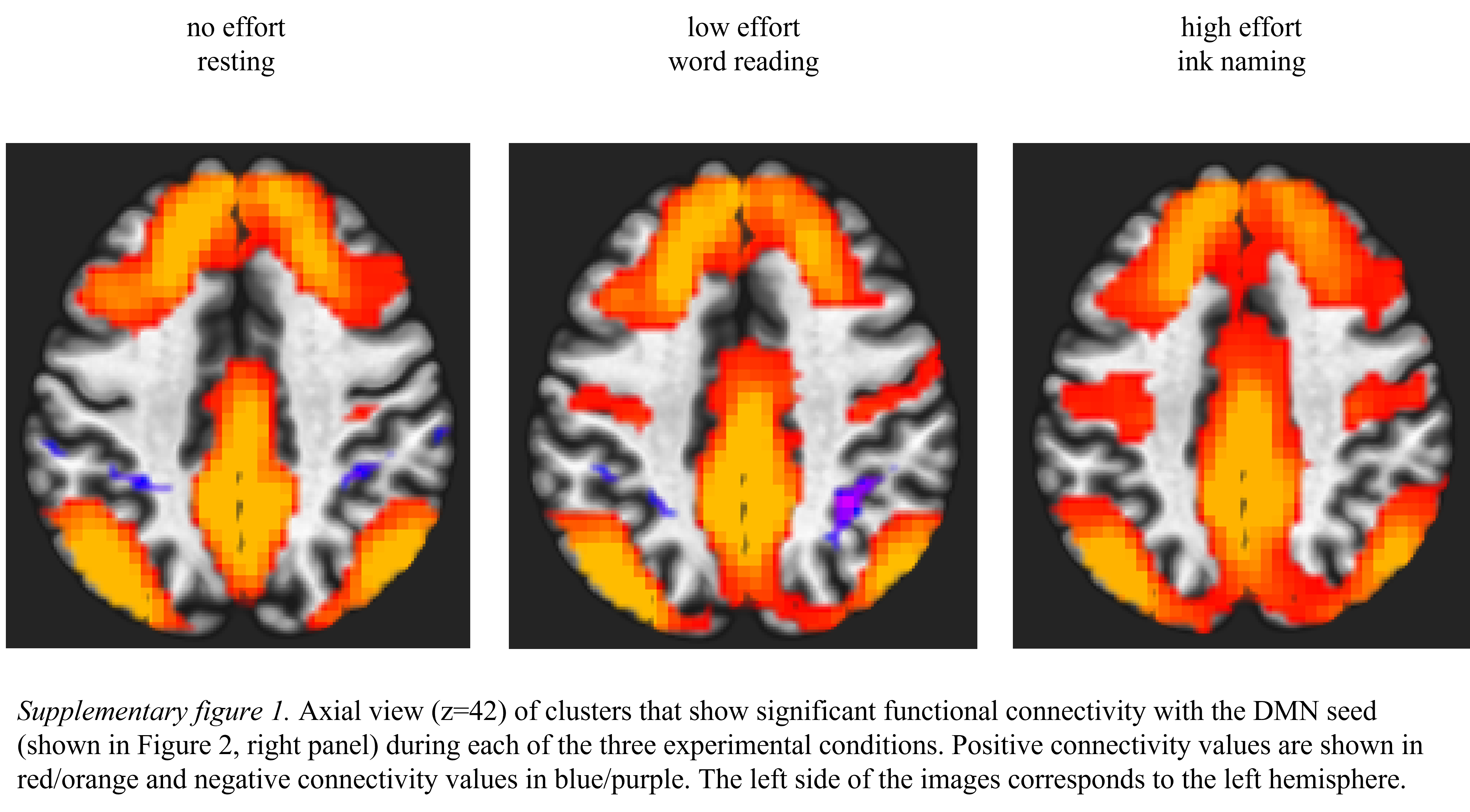

Supplement: Supplementary file 1 — Supplementary Information 1. [file 41598_2022_10289_MOESM1_ESM.png]
